# Recent Advances in Adipose Tissue Dysfunction and Its Role in the Pathogenesis of Non-Alcoholic Fatty Liver Disease

**DOI:** 10.3390/cells10123300

**Published:** 2021-11-25

**Authors:** Xiaoxiao Wang, Huiying Rao, Feng Liu, Lai Wei, Honggui Li, Chaodong Wu

**Affiliations:** 1 Beijing International Cooperation Base for Science and Technology on NAFLD Diagnosis, Beijing Key Laboratory of Hepatitis C and Immunotherapy for Liver Diseases, Peking University Hepatology Institute, Peking University People’s Hospital, Beijing 100044, China; wangxx0635@163.com (X.W.); rao.huiying@163.com (H.R.); liu1116m@sina.com (F.L.); 2 Beijing Tsinghua Changgung Hospital, Tsinghua University, Beijing 102218, China; weilai@mail.tsinghua.edu.cn; 3 Department of Nutrition, Texas A&M University, College Station, TX 77843, USA; hgli@ag.tamu.edu

**Keywords:** obesity, adipose dysfunction, inflammation, crosstalk, nonalcoholic fatty liver disease (NAFLD)

## Abstract

Obesity is a serious ongoing health problem that significantly increases the incidence of nonalcoholic fatty liver disease (NAFLD). During obesity, adipose tissue dysfunction is obvious and characterized by increased fat deposition (adiposity) and chronic low-grade inflammation. The latter has been implicated to critically promote the development and progression of NAFLD, whose advanced form non-alcoholic steatohepatitis (NASH) is considered one of the most common causes of terminal liver diseases. This review summarizes the current knowledge on obesity-related adipose dysfunction and its roles in the pathogenesis of hepatic steatosis and inflammation, as well as liver fibrosis. A better understanding of the crosstalk between adipose tissue and liver under obesity is essential for the development of new and improved preventive and/or therapeutic approaches for managing NAFLD.

## 1. Introduction

### 1.1. Obesity and Associated Metabolic Diseases

Obesity is a disease characterized by increased body fat mass or body mass index (BMI, body weight divided by the height in meters squared (kg/m^2^)) [1]. Based on BMI, individuals can be divided into six groups including low weight (<18.5 kg/m^2^) normal weight (18.5–24.9 kg/m^2^), overweight (25.0–29.9 kg/m^2^), class 1-obesity (30.0–34.9 kg/m^2^), class 2-obesity (35.0–39.9 kg/m^2^) and class 3-obesity (≥40 kg/m^2^) [2]. In general, obesity is considered one of the most common diseases in the 21st century [3]. According to the data from the WHO, more than 1.9 billion individuals were overweight, among whom 650 million were obese worldwide [4]. The reasons for the development of obesity are multifactorial, including genetics, increased food availability and density, decreased physical activity, and changes in environmental and socio-economic factors [5,6].

Epidemiological data demonstrate that the prevalence rates of metabolic syndrome (MS) in populations vary from 20% to 45%. As the most common phenomenon of MS [1], obesity is highly associated with the development of various metabolic and non-metabolic diseases, such as nonalcoholic fatty liver disease (NAFLD), type 2 diabetes mellitus (T2DM), asthma, cardiovascular diseases (CVD), and certain forms of cancer [7,8]. NAFLD is a common metabolic disease and the incidence of NAFLD increases while BMI is increasing [9]. For instance, researchers demonstrated that in nonobese populations, the prevalence rates of steatosis and steatohepatitis are approximately 15% and 3%, respectively, whereas in class 1- and 2-obesity individuals approximately 65% and 20%, and in class 3-obesity people approximately 85% and 40% [10,11,12,13,14]. This review, along with many others, strongly implicates a critical role for obesity in initiating or exacerbating the pathogenesis of NAFLD.

### 1.2. Adipose Tissue Dysfunction in the Pathogenesis of NAFLD

In humans, there are two different types of adipose tissues, brown adipose tissue (BAT) and white adipose tissue (WAT) [15]. While WAT is the predominant form of adipose tissue found in adults and is responsible for fat storage [15], BAT is mainly located in small and discrete regions of newborns and is responsible for heat production. Furthermore, recent evidence indicates that active BAT can be found in specific adipose depots in adult humans, some of which reveal a thermogenic potential [16].

Obesity is mainly characterized by increased size in WAT and significant changes in the secretory function [17]. Indeed, abnormalities in the secretory function of WAT are common during obesity. WAT secretes adipokines and various bioactive substances (e.g., leptin, interleukin (IL)-6, IL-1β, tumor necrosis factor (TNF)-α), which participate in a series of pathophysiological activities, including glucose and lipid metabolism, inflammatory responses, blood pressure regulation and food ingestion [18,19]. For instance, leptin, as a WAT-secreted hormone whose levels are positively correlated with body weight and fat mass, is shown to also play a critical role the pathophysiology of NAFLD. In a population-based study involving 1610 subjects with NAFLD from a total of 4571 subjects, the circulating levels of leptin for normal, mild, moderate and severe steatosis were 10.7 ± 0.3 ng/mL, 12.1 ± 0.7 ng/mL, 15.6 ± 0.8 ng/mL, 16 ± 1.0 ng/mL, respectively. This suggests a strong positive association between leptin and the severity of NALFD [20]. However, this association does not necessarily indicate a causal or pathogenic role for leptin in causing hepatic steatosis. This is particularly important while considering that obese human subjects with elevated levels of leptin generally reveal leptin resistance. In contrast, a previous study by Canbakan et al. indicates a preventive effect of leptin against progressive liver injury in NAFLD. Because of this, the precise role of leptin in NAFLD remains to be elucidated. Similarly, adiponectin, as an essential adipocyte-secreted hormone that critically regulates systemic insulin sensitivity, has also been implicated to play a role in NAFLD pathophysiology [21]. In particular, the results from the study by Kaser et al. suggest that reduced hepatic expression of adiponectin, along with adipoRII (adiponectin receptor II), appear to be of pathophysiological relevance in NALFD [22]. A recent study further validated that treatment with adiponectin is capable of ameliorating hepatic steatosis associated with type 1 diabetes in rats [23]. Considering that adiponectin has broad effects on metabolic tissues (e.g., skeletal muscle) and systemic insulin sensitivity, the extent to which the direct actions of adiponectin on the liver contribute to the anti-NAFLD effect of adiponectin remains to be determined.

### 1.3. Differential Functions of Adipose Tissue Depots in Pathogenesis of NAFLD

While WAT is considered a heterogenous tissue/organ, different WAT depots have been shown to display different functions. This is particularly true with regard to the roles of subcutaneous fats versus visceral fats in regulating systemic insulin sensitivity. As a primary target of thiazolidinediones (TZDs), subcutaneous WAT, but not visceral fat, appears to largely account for the insulin-sensitizing effect of TZDs. Given that insulin sensitivity is a key factor regulating NAFLD pathogenesis, differences in roles for WAT depots in NAFLD have been proposed. Indeed, the outcome of a recent study involving human subjects with NAFLD and T2DM indicates that visceral WAT is a major determinant of the severity of hepatic steatosis and liver stiffness [24]. Consistently, removal of epididymal visceral WAT prevented obesity-induced insulin resistance (IR) and hepatic steatosis in mice [25], whereas removal of mesenteric WAT aggravated NAFLD. Furthermore, as supported by various lines of evidence from rodent models, mesenteric fats are shown to be closely associated with the degrees of NAFLD phenotype in mice with high-fat diet (HFD)-induced NAFLD [26]. Mechanistically, mesenteric fats likely function through regulating their own inflammatory status and maintaining intestinal barrier integrity to regulate NAFLD phenotype.

It is also important to note that during obesity both WAT hypertrophy and hyperplasia occur simultaneously. However, unlike WAT hypertrophy, which is commonly associated with increased inflammation, WAT hyperplasia appears to be protective. As supporting evidence, hyperplasia in obese women is predominant in the subcutaneous fat depot whereas WAT hypertrophy is observed both in the omental and subcutaneous compartments [27]. Accordingly, the role of WAT hyperplasia in NAFLD is likely linked to the function of subcutaneous fats in improving systemic insulin sensitivity.

### 1.4. Brown Adipose Tissue and NAFLD

Brown adipose tissue (BAT) contributes to energy expenditure in the form of heat. In recent years, lots of investigators have studied the relationship between BAT and the development and progression of NAFLD. Compared with individuals without NAFLD, NAFLD patients show lower BAT activity, which is inversely associated with liver fat content [28]. Furthermore, a study originated from a large population in the United States demonstrates that the activity of BAT is associated with the improvement of central obesity and metabolic disorders (including blood glucose and lipids, liver fat deposition and T2DM). Based on this, there might be an inevitable relationship between the BAT and the remission of NAFLD [29]. The underlying mechanisms by which BAT is involved in liver metabolism include: (1) decreasing the levels of circulating fatty acids to reduce liver lipotoxicity; (2) secreting cytokines to regulate liver lipid metabolism; and (3) interacting with other organs to reduce liver lipid deposition [30,31]. For instance, Li P et al. demonstrated that BAT transplanted from healthy mice to diabetic mice inhibits the expression of NOX4 protein by upregulating miR-99a, thereby improving the abnormal glucose and lipid metabolism in the liver [32]. Because of this, BAT may also be a viable target for managing NAFLD.

## 2. Pathophysiology of NAFLD during Obesity

### 2.1. The Prevalence of NAFLD

NAFLD is defined as the presence of liver steatosis (≥5%), and in the absence of other liver diseases, such as chronic viral hepatitis, significant alcoholic consumption, drug-induced liver injury, autoimmune hepatitis, and hereditary metabolic liver disease (e.g., hemochromatosis, Wilson’s disease) [33]. When the liver displays overt inflammatory damage, NAFLD progresses from simple steatosis to steatohepatitis (NASH), which may eventually lead to the development of liver fibrosis, cirrhosis and hepatocellular carcinoma (HCC) [34]. It is estimated that 24% of the general population of the whole world has NAFLD [33]. NAFLD is highly prevalent in many continents and is shown at rates of 31% in South America, 32% in the Middle East, 27% in Asia, 24% in the USA and 23% in Europe [33]. The prevalence rate of NAFLD is paralleling with the increase in the incidence of obesity worldwide [33,35]. NAFLD is estimated to become the leading cause of liver failure and transplantation worldwide [36].

### 2.2. Two Hit or Multiple Hit Mechanisms of NAFLD

The “Two-hit” theory is commonly used to explain the pathogenesis of NAFLD, which highlights that, in the setting of NAFL (steatosis alone), the liver receives the ‘second hit’ from other factors, which contribute to NASH occurrence and progression. However, this theory cannot fully explain the pathogenesis of NAFLD [37]. In particular, the mechanisms leading to NASH and its clinical features are highly heterogeneous. Numerous metabolic and signaling molecules are shown to be involved in the progression of NASH [38]. Accordingly, many researchers proposed the “multiple-hit” theory for NAFLD. The excessive amount of metabolic energy substrates (hepatic or extrahepatic sources) appears to serve as regulators for the generation of lipotoxic species. In the setting of liver inflammation, it is overwhelming for the liver to handle the toxic lipid species, which induce hepatocellular injury and death, liver fibrosis, cirrhosis and HCC [39,40]. In addition, oxidative stress, inflammatory molecules, apoptotic damages and the consequent compensatory regeneration also serve as regulators in “multiple-hit” theory [41]. Therefore, the fact that liver fibrosis or cirrhosis occurs in either presence or absence of steatosis convincingly supports the notion that steatosis is not the sole causal factor of the development and progression of liver inflammation, which is a key characteristic of NASH and commonly results in liver fibrogenic damages.

### 2.3. Dysregulation of Fat Metabolism in the Liver

The outcomes of hepatic fat metabolic fluxes determine whether the liver deposes or exports fats. In general, increased fluxes for lipogenesis and fatty acid uptake by hepatocytes, along with decreased fluxes through fatty acid oxidation (FAO) and the release of very-low-density lipoproteins (VLDLs) in hepatocytes, cause or exacerbate hepatic steatosis. As such, how fat metabolic fluxes are regulated is of particular importance in the development and progression of NAFLD.

In hepatocytes, *de novo* lipogenesis (DNL) is stimulated in response to nutrient sufficiency. Metabolites of glycolysis serve as critical substrates for free fatty acids (FAs)and then triglycerides. The latter are packaged into VLDLs and released into the circulation as endogenous fats, which in turn are stored in WAT as energy to be used under fasted states. Of note, the product of the key regulatory step of lipogenesis catalyzed by acetyl-CoA carboxylase (ACC) generates malonyl-CoA, which is a powerful inhibitor of carnitine palmitoyl transferase 1a (CPT1a), the key enzyme regulating the rate-limiting step of FAO. Indeed, during fasted states, DNL is decreased, resulting in decreased production of malonyl-CoA. This releases the inhibitory effect on FAO. Meanwhile, increased WAT lipolysis releases FAs to the circulation for uptake by the liver and other metabolic tissues, e.g., skeletal muscle. As a transcription factor, peroxisome proliferator-activated receptor α (PPARα) is abundantly expressed in the liver and plays an important role in stimulating FAO.

As reviewed by many scientists, dysregulated hepatic fat metabolism accounts for the development and progression of NAFLD. More importantly, WAT dysfunction contributes to dysregulation hepatic fat metabolism, which is highlighted in the following sections.

### 2.4. Pathogenesis of Liver Inflammation

Inflammation is a critical pathophysiological process in liver injury, and the severity of inflammation that is correlated with steatosis could increase the risk of NAFLD progression [42,43]. Furthermore, during NASH, there are two important features of inflammation, including an increased number of immune cells and pro-inflammatory activation of these immune cells [44]. Both the cells residing in the liver, e.g., Kupffer cells, and cells recruited from extrahepatic sources, e.g., monocytes and natural killer cells, can produce pro-inflammatory factors, which contribute to apoptosis or necrosis of hepatocytes [45]. On the basis of liver inflammation, extrahepatic inflammatory cells can be recruited to the inflammation sites through the interactions between the chemokines/cytokines and their ligands [46]. In addition, inflammasome activation in the liver also leads to the expression of pro-inflammatory cytokines, e.g., IL-1β, IL-18, and promotes apoptosis through caspase-1 activation. Inflammation in adipose tissue, particularly visceral adipose tissue (VAT), has the potential to be expanded to the liver and is associated with NAFLD. For instance, in HFD mouse models, inflammatory signals shift from adipose to the liver and influence NASH progression [47]. Furthermore, during obesity, macrophage pro-inflammatory activation in adipose tissue is responsible for the early stages of NASH and IR in the liver and other peripheral tissues. However, deletion of adipose macrophages did not reverse the later phase (inflammation and fibrosis) of NAFLD once inflammation was established [48,49].

During obesity, an excessive amount of fat deposition in hepatocytes, largely due to increased DNL and/or fat overflow from WAT to the liver, induces TNFα production and the release of reactive oxygen species (ROS), which in turn contribute to the development and progression of liver inflammation and NASH phenotype [50]. In addition, excessive deposition of FAs also induces lipotoxicity, which promotes apoptosis, inflammation, fibrogenesis, and the progression to NASH [39]. Mechanistically, palmitate, as a major pro-inflammatory FA, has direct effects on causing mitochondrial dysfunction (increased ROS production) and activating pro-inflammatory signaling (JNK1 phosphorylation) [51]. Consistent with this mechanism, palmitate treatment also has been shown to cause increased pro-inflammatory signaling in several other types of liver cells, including mouse primary hepatocytes and macrophages [52,53]. More recently, Yu et al. showed that palmitate stimulated mitochondrial dysfunction, which produced mtDNA. The latter serves as a mediator underlying how pro-inflammatory signals generated by hepatocytes in response to palmitate treatment act on macrophages to stimulate stimulator of interferon genes (STING) activation, thereby macrophage pro-inflammatory activation [54]. This adds new insights as to how hepatocyte fat deposition critically contributes to the development of NAFLD in mouse models and in humans [55,56]. However, it remains to be explored the proportional contribution of dysfunctional adipose tissue-driven delivery of an excessive amount of fats to hepatocytes versus DNL-driven fat deposition and related excessive fatty acid oxidation to increased hepatocyte pro-inflammatory responses.

In hepatocytes, there also exist various immune molecules and signaling pathways. For example, during NASH, hepatocyte inflammasome activation is an important link between the initial metabolic stress and subsequent hepatocyte death [57]. Stressed hepatocytes released much more extracellular vesicles (EVs), which in turn promoted macrophage accumulation in the liver during NASH. This indicates that complex crosstalk between hepatocytes and immune cells plays a pivotal role in liver inflammation [58].

Interestingly, there is also growing evidence mechanistically illustrating exactly how adipose tissue dysfunction, more specifically, adipocyte death, causes NAFLD. For instance, Kim SJ et al. demonstrated that adipocyte death selectively and specifically in mice induced adipose tissue macrophage infiltration and subsequent liver lipotoxicity, inflammation and damage through activating CCR2^+^ macrophages [59]. Therefore, liver inflammation is a complex pathophysiological phenomenon involving various types of liver cells.

### 2.5. Hepatocyte Fat Deposition and Regulation of Fat Metabolism

While *de novo* synthesis of FAs occurs in the cytosol, FAO takes place in mitochondria and peroxisomes of hepatocytes. Acetyl-CoA and nicotinamide adenine dinucleotide phosphate (NADPH) are substrates for lipogenesis. As the most important enzyme in the regulation of lipogenesis, ACC catalyzes the generation of malonyl-CoA. Subsequently, fatty acid synthetase (FAS) catalyzes seven steps in the fatty acid synthesis process, resulting in the synthesis of palmitate from malonyl-CoA and acetyl-CoA [60].

Lipogenesis is subject to regulation by nutritional and hormonal signals. For example, glucose and insulin stimulate glycolysis to provide substrates for lipogenesis and promote ACC and FAS gene expression. Enhancing glycolysis in the liver, in particular at the step of glucose phosphorylation, promotes lipogenesis [60]. Furthermore, dietary fructose increases hepatic DNL via stimulating sterol regulatory element-binding protein-1c (SREBP-1c) expression [61]. During obesity, there appears to exist “selective IR” in the liver. On the one hand, impaired insulin signaling fails to inhibit forkhead box O1 (FoxO1), thereby losing a suppressive effect on hepatic gluconeogenesis. On the other hand, the insulin-signaling pathway activates the mammalian target of rapamycin complex 1 (mTORC1) to increase lipogenesis in the liver [62]. In addition, both the liver and adipose tissue exist certain mechanisms through which fat acid synthesis is promoted. As reviewed elsewhere, a number of nuclear receptors, e.g., PPARγ, are shown to promote fat synthesis in both the liver and adipose tissue [63,64,65]. It should be noted that adipose tissue-derived fatty acids, additional to fatty acids produced by hepatocyte lipogenesis, are also important sources to provide substrates for the formation of triglycerides and lipid droplets, leading to hepatocyte fat deposition [66,67]. This, indeed, highlights the importance of obesity-associated adipose dysfunction in the development and progression of hepatic steatosis.

## 3. Dysfunctional Adipose-Liver Crosstalk in the Pathogenesis of NAFLD

**Figure 1:**
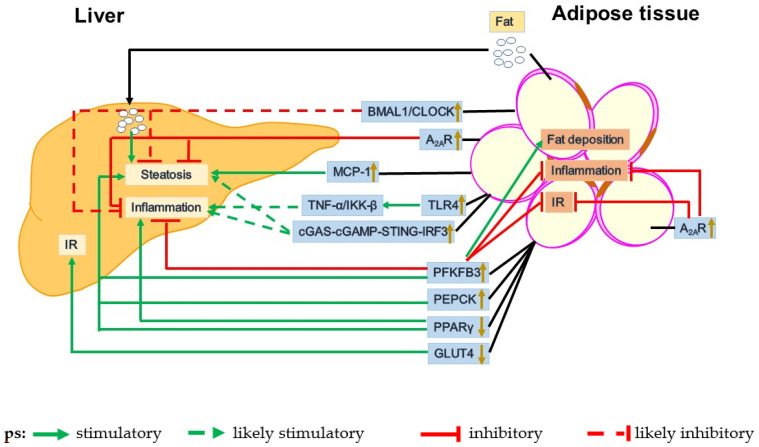
Dysfunctional crosstalk between adipose tissue and the liver in the pathogenesis of NAFLD. A number of signaling pathways and factors from the adipose tissue function to induce liver dysfunction. For instance, increased expression of PFKFB3 and PEPCK or decreased expression of PPARγ and GLUT4 promotes liver steatosis, inflammation and/or IR whereas overexpression of PFKFB3 in adipose tissue inhibits liver inflammation. Additionally, upregulated cGAS-STING-IRF3, TLR4 and/or MCP1 in adipose tissue, appears to exacerbate liver inflammation and steatosis. However, upregulated BMAL1/CLOCK functions to protect against liver steatosis and inflammation. In addition, fats released from the adipose tissue are transported to the liver and aggravate liver steatosis.

As mentioned above, dysfunctional adipose tissue promotes the pathogenesis of NAFLD at least through increasing the delivery of fats and adipokines to the liver to increase hepatic steatosis and inflammation. As such, dysfunctional adipose-liver crosstalk serves as a key mechanism underlying the development and progression of NAFLD. The data from human studies have suggested that adipose tissue-derived fats contribute to liver fat deposition. For example, in NAFLD patients, peripheral fats stored in adipose tissue are a main source of plasma non-esterified fatty acids (NEFAs), which flow to the liver to promote TG accumulation in hepatocytes [67]. This exemplifies the role of dysfunctional adipose tissue-liver crosstalk in the pathophysiology of NAFLD. Taking advantage of genetically modified mice, researchers have been able to generate compelling data to elucidate the mechanistic insights concerning the role of dysfunctional adipose tissue in NAFLD pathophysiology (Figure 1).

### 3.1. GLUT4

As a key molecule of glucose metabolism, glucose transporter 4 (GLUT4) medicates insulin-stimulated glucose uptake in adipocytes through its translocation from intracellular storage sites to the cell membrane rapidly [68]. However, under the state of IR, such as T2DM and obesity, the expression of GLUT4 in adipose tissue was decreased significantly [69]. In adipocyte GLUT4 knockout mice, researchers performed hyperinsulinemic-euglycemic clamp studies involving assessment of 2-deoxyglucose uptake into individual tissues and measured insulin function on liver glucose production. Their major findings indicated that hepatic glucose production was normal at baseline, but was not responsive to insulin [70]. Therefore, selective downregulation of GLUT4 in adipocytes caused glycometabolism dysregulation and IR in the liver.

### 3.2. PEPCK

The enzyme phosphoenolpyruvate carboxykinase (PEPCK) plays a crucial role in gluconeogenesis in the liver and glyceroneogenesis in adipose tissue [71,72]. Under a normal diet, adipose tissue-specific PEPCK-overexpressing transgenic mice revealed significant increases in body weight, adipocyte size and fat mass, as well as significantly decreased FFAs in circulation, relative to control mice. However, liver steatosis was not altered and insulin sensitivity of whole-body was preserved [73]. In addition, upon HFD feeding, adipose tissue-specific PEPCK-overexpressing mice showed severe obesity, hyperinsulinemia and IR, along with significantly increased hepatic fat deposition and triglyceride content [74]. In terms of the mechanisms underlying hyperglycemia and hyperinsulinemia induced by PEPCK overexpression, it appeared that lipid accumulation in the liver functioned to inhibit glucose metabolism and decrease hepatic insulin extraction [75,76]. Because of this, overexpression of PEPCK in adipose tissue caused glycometabolism dysregulation, which contributed to the development of hepatic steatosis.

### 3.3. PFKFB3

6-phosphofructo-2-kinase/fructose-2,6-biphosphatase 3 (PFKFB3) encodes inducible 6-phosphofructo-2-kinase (iPFK2), whose product fructose 2,6-bisphosphate (F26P2) is the most powerful activator of 6-phosphofructo-1-kinase (6PFK1, a key glycolytic enzyme) [77,78]. It was previously shown that adipose tissue-specific PFKFB3-overexpression in mice significantly increased liver weight, hepatic steatosis, and hepatic expression of lipogenesis genes such as ACC1, FAS, and SREBP1c. However, adipose tissue-specific PFKFB3-overexpression decreased hepatic phosphorylation of nuclear factor kappa-B (NFκB) and expression of pro-inflammatory cytokines [79]. Consistently, treatment of mouse primary hepatocytes with conditioned medium of PFKFB3/iPFK2-overexpressing adipocytes caused a significant increase in fat deposition, but significant decreases in hepatocyte ROS production, NFκB phosphorylation, and pro-inflammatory cytokines production [79]. In addition, Huo et al. postulated that dysregulated liver glucose metabolism in HFD-fed heterozygous PFKFB3-disrupted mice was likely due to a secondary effect of PFKFB3 disruption in adipose tissue [80]. Therefore, overexpression of PFKFB3 in adipose tissue disassociated steatosis, inflammation and IR in the liver.

### 3.4. PPARγ

Peroxisome proliferator-activated receptor γ (PPARγ) is expressed abundantly in adipocytes and functions as a transcriptional regulator to play an essential role in lipid metabolism [64,81]. It was shown that adipose-specific-PPARγ KO mice exhibited significant increases in liver weight, lipid deposition, glycogen content and metabolism, and CD36 and GLUT4 expression. This phenotype was accompanied by increased hepatic PPARγ and adiponectin expression, along with overall improvement in systemic insulin sensitivity and HFD-induced obesity [65]. He et al. showed that targeted disruption of PPARγ in adipose tissue of HFD mice induced hyperlipidemia, liver steatosis, and reduction of circulating leptin and ACRP30, however, these mice gained less body weight and was accompanied with increased hepatic IR [81]. To be noted, PPARγ deficiency in adipose tissue may drive macrophage polarization toward M1 pro-inflammatory activation in the liver [63]. Collectively, PPARγ disrupted in adipose tissue contributed to liver steatosis and inflammation progression. Furthermore, it is worth noting that PPARγ activation brings about insulin-sensitizing effects, along with the unwanted effect on increasing adiposity [82]. This raises a concern on the reliability or sustainability in using PPARγ agonist(s) in managing NAFLD.

## 4. Obesity-Associated Adipose Tissue Dysfunction

### 4.1. Adipose Tissue Inflammation and Its Regulation by Macrophages

Adipose tissue contains lots of immune cells, which are responsible for maintaining the bio-physiological function of adipocytes [7]. A significant body of literature has demonstrated obesity-associated inflammation as a causal factor of IR and metabolic dysregulation [83,84]. For instance, obesity or nutrition stress, e.g., nutrient overload or unhealthy nutrition, triggers or exacerbates WAT inflammation that is characterized by macrophage infiltration and adipose tissue dysfunction that contributes to the pathogenesis of systemic IR and T2DM [80,85,86,87,88,89], as well as NAFLD and CVD [59,79,81,90]. It is now well-accepted that during obesity, adipose tissue undergoes radical changes of the immune cell composition, which are accompanied by increased production of various inflammatory factors, and activation of pro-inflammatory signaling pathways [91,92]. These changes, in turn, function to enhance lipolytic activity and decrease insulin sensitivity in adipocytes [93]. As it is documented elsewhere, the triggers of obesity-associated inflammation include various metabolites, adipocyte death, hypoxia, intestinal antigens, and mechanical transmission resulting from the interaction between the extracellular matrix (ECM) and adipocytes [7].

During obesity, macrophages largely account for the initiation or exacerbation of chronic inflammation in expanded adipose tissue, and the pro-inflammatory state of adipose tissue is associated with macrophage activation status [85,89]. In particular, dysregulated macrophage functional plasticity and versatility (polarization) are key components through which inflammation is initiated or exacerbated in metabolic tissues, e.g., WAT and the liver. According to prior research, within macrophages, peroxisome proliferator-activated receptor gamma and delta (PPARγ/δ) are key transcription factors that stimulate macrophage anti-inflammatory activation [94,95]. This is significant because myeloid cell-specific disruption of PPARγ and/or PPARδ increases pro-inflammatory activation of adipose tissue macrophages and exacerbates obesity-associated IR [95]. In contrast, the effect of PPARγ activation on reversing HFD-induced IR is mediated, at least in part, by stimulation of anti-inflammatory activation of macrophages in adipose tissue [89]. Macrophage activation status is also regulated by Toll-like receptor 4 (TLR4), c-Jun N-terminal kinase (JNK), and STING, such that their myeloid cell-specific disruption protects mice from diet-induced IR and WAT and liver inflammation and metabolic dysregulation [56,96,97]. Recent advances pertinent to how these regulators alter macrophage function in the context of regulating WAT inflammation and NAFLD pathogenesis have been further discussed in the following sections.

### 4.2. Uncoupling Fat Deposition and Inflammation

Although it is commonly assumed that a vicious cycle exists within glycolipid metabolism and inflammation, mounting evidence indicates that fat deposition, inflammation and IR can be dissociated in obesity [77,78,79,80,81,98]. For instance, PFKFB3/iPFK2 plays a unique role in dissociating adiposity from adipose tissue inflammation and insulin sensitivity [79,80]. In the pathway of glucose metabolism, PFKFB3 is highly expressed in adipose tissue and encodes for iPFK2 [77]. PFKFB3/iPFK2 generates F26P2, which in turn activates 6PFK1 to promote glycolysis. This pathway is also involved in adipocyte lipogenesis and TG synthesis [77,78]. Disruption of PFKFB3/iPFK2 decreases HFD-induced obesity, but exacerbates adipose tissue inflammation, which contributes to an increase in the severity of IR [80]. In contrast, overexpression of PFKFB3/iPFK2 in adipose tissue exacerbates fat deposition but decreases systemic IR and inflammatory responses [79]. In addition, in an HFD-mouse model, liver steatosis, inflammation and IR can be dissociated in response to supplementation of palmitoleate (a monounsaturated fatty acid), which increases fat deposition in hepatocytes, but protects against the inflammatory response in both hepatocytes and macrophages [98,99]. In a recent study by Guo et al., it has been further validated that the PFKFB3 in adipocytes is needed, on the one side, for HFD feeding to induce hepatic steatosis, and, on the other side, for PFKFB3-driven adipocyte factors to suppress distal inflammation in the liver [100]. Clearly, PFKFB3 signifies how adipose tissue plays an essential role in regulating hepatic steatosis and liver inflammation, two key aspects of NAFLD/NASH.

### 4.3. Circadian Dysregulation

Circadian clocks located in various cells and tissues regulate the daily rhythms of our body. Mounting evidence indicates that circadian clocks also play important roles in the regulation of inflammation, metabolism and other pathophysiological processes [101,102]. However, it is unclear how circadian clock disruption results in metabolic disorders. In the HFD-mouse model, circadian clock disruption is shown to induce the activation of pro-inflammatory macrophages and inflammatory signaling pathways, which lead to the enhancement of adipose tissue inflammation, hyperglycemia and IR and the increase of body weight [103]. Moreover, nutrition stress causes the activation of pro-inflammatory signaling (JNK1 and NFκB), which leads to the dysregulation of the circadian clock conversely [103]. Polymorphisms of the clock genes Bmal1 and clock are associated with obesity and T2DM. Furthermore, HFD-Bmal1 knockout mouse displayed significant fat accumulation and elevated body fat compared with the control mouse. In contrast, constitutive expression of Bmal2 gene (Bmal1 and Bmal2 is a circadian paralogous pair) was shown to rescue the phenotype of “clockless” Bmal1 knockout mouse nearly to the normal level, but it was unable to rescue the phenotype of isolated WAT from Bmal1 knockout mice [104]. Per3, another circadian clock gene, also has an important role in mediating adipogenesis. In endogenous adipocyte precursor cells (APCs), deletion of Per3 promotes adipogenesis by a clock output pathway in which PER3 and BMAL1 directly regulate Kruppel-like family member Klf15 expression [105]. Accordingly, it is very likely that circadian dysfunction plays an important role in rodent model systems in the context of regulating adipose tissue function.

Various studies involving human subjects suggest a close link between circadian dysregulation and NAFLD. In particular, disturbed lifestyles including night shift work, social jet lag, wrong-time feeding, and sleep disorders that result in circadian clock dysregulation are shown to be associated with increased incidence of NAFLD [106]. Of note, multiple studies also have validated that circadian dysregulation is associated with an increased risk of obesity and/or metabolic syndrome [107,108,109], which all are factors that cause or exacerbate aspects of NAFLD. Considering that obesity increases the incidence of NAFLD by 7 to 10 folds, it is very plausible that circadian dysregulation-driven adipose dysfunction contributes to the development and progression of NAFLD; although the outcomes of human studies are not able to establish a cause-and-effect relationship.

### 4.4. Adipocyte and Macrophage Inflammatory Signaling

As mentioned above, macrophages have been implicated to play a critical in the pathogenesis of adipose tissue inflammation, which in turn contributes to the development and progression of NALFD. In this section, recent advances in several key signaling pathways that regulate adipose tissue inflammation are summarized.

#### 4.4.1. TLR4

TLR4 belongs to the pattern recognition receptor (PRR) family and functions to regulate the innate immune system. Much evidence has demonstrated critical roles for TLR4 in regulating obesity-associated inflammation and systemic insulin resistance [110,111]. The latter aspects, as mentioned above, are well accepted to contribute to the pathogenesis of NAFLD. Indeed, a detrimental role for TLR4 in NAFLD is supported by the finding that TLR4 deficiency protected mice from HFD-induced hepatic steatosis and inflammation [112]. Considering that TLR4 is expressed in multiple types of cells, an important question concerning NAFLD pathogenesis is whether and how the TLR4 in different cell types contributes to the development and progression of hepatic steatosis and inflammation. Using mice in which TLR4 has disrupted only hepatocytes, Li et al. demonstrated that TLR4 deficiency in hepatocytes alleviated the effect of HFD on increasing liver weight and hepatic levels of triglycerides [113]. Using chimeric mice in which TLR4 was disrupted only in hematopoietic cells, Saberi et al. demonstrated that TLR4 deficiency only in hematopoietic cells also decreased hepatic levels of triglycerides and protected mice from HFD-induced liver inflammation and insulin resistance [114]. To be noted, upon HFD feeding, chimeric mice whose TLR4 was disrupted only hematopoietic cells also displayed adipose tissue inflammation. The latter is thought to contribute to NAFLD phenotype via increased delivery of inflammatory adipokines to the liver. Moreover, adipocytes, *per se*, reveal increased pro-inflammatory responses in a TLR4-dependent manner upon treatment with free fatty acids (mixture of palmitate and oleate) [111]. This led to a simple postulation that the TLR4 in adipocytes also has a detrimental role in the pathogenesis of NAFLD through enhancing the delivery of pro-inflammatory adipokines, generated in response to increased adipose tissue inflammation, to the liver. However, the study by Tao showed that adipocyte-specific TLR4 deletion in mice significantly increased the severity of HFD-induced hepatic steatosis while improving systemic insulin sensitivity [115]. Given this, the role for TLR4 in regulating adipose tissue inflammation and metabolic dysregulation in relation to NAFLD pathophysiology appears to be complex and needs to be further studied.

#### 4.4.2. MCP1

Monocyte chemotactic protein 1 (MCP1), a member of the CC chemokine family, has been implicated in several chronic inflammatory diseases and associated with obesity in humans [116]. Under obese conditions, adipose expression of MCP1 is increased in both human subjects and rodents (*db/db* mice and mice with HFD-induced obesity) [117]. Because of this, various studies have investigated the pathophysiological role of MCP1 in regulating WAT inflammation and metabolic regulation. For instance, the study by Kamei et al. showed that adipocyte-specific MCP1 overexpression in mice significantly increased adipose tissue macrophage inflammation, which was accompanied by increased WAT inflammation and systemic insulin resistance [118]. Of note, adipocyte-specific MCP1 overexpression also increased hepatic glucose production and decreased hepatic insulin signaling. This implies changes in hepatic events that were associated with NAFLD; although NAFLD aspects were not examined in the study. In contrast, the study by Kanda et al. showed that MCP1 disruption alleviated HFD-induced adipose tissue inflammation and systemic insulin resistance in mice with genetic or diet-induced obesity [119]. Concurrently, MCP1 disruption also significantly decreased the severity of HFD-induced hepatic steatosis, likely through mechanisms involving down-regulation of hepatic SREBP1c. The latter is a key regulator whose activation stimulates the transcription of genes for key lipogenic enzymes. Because increased NAFLD phenotype was accompanied by decreased WAT inflammation. It is likely that MCP1 serves as a mediator linking WAT dysregulation and NAFLD phenotype. Interestingly, a recent study by Baeck et al. showed that pharmacological inhibition of MCP1 significantly alleviated the NASH phenotype in mice. This effect was attributable to, in large part, the reduction of hepatic macrophage infiltration [119,120]. However, it is not clear the extent to which the treatment also decreased WAT inflammation, thereby contributing to the alleviation of the NASH phenotype; although MCP1 inhibition is expected to also decrease WAT inflammation.

#### 4.4.3. JNK

Recent studies in rodents suggest that JNK is a key player in regulating adipose tissue inflammation and obesity [121]. Upon JNK1 disruption in obese mice, the body weight, adipocyte size, and adiposity were decreased significantly. In addition, blood glucose and insulin concentrations were also decreased markedly. These results indicated that JNK1 deficiency protected mice from obesity-induced IR [121]. Consistently, in HFD mice in which JNK1 and JNK2 were both disrupted in adipose tissue, body weight, fat mass, the size of the adipocytes, and macrophage infiltration in adipose tissue were all reduced significantly [122]. Simultaneously, this line of transgenic mice was resistant to the deleterious impact of an HFD on liver steatosis and gluconeogenesis [122]. Of note, the most important finding was that adipocyte-specific deletion of JNK1 improves liver insulin sensitivity and liver steatosis [123]. As such, the JNK1 in adipose tissue appears to play a detrimental role in the progression of liver steatosis and IR.

#### 4.4.4. A_2A_R

Adenosine 2A receptor (A_2A_R) is an anti-inflammatory protein and plays a protective role in obesity-associated adipose tissue inflammation [53,124]. In HFD-fed wild-type mice, A_2A_R was present predominantly in macrophages of adipose tissue and its expression in adipose tissue was increased significantly [124]. However, in response to A_2A_R disruption, HFD-fed mice displayed significant increases in adipose tissue inflammation and IR, including enhanced pro-inflammatory signaling and pro-inflammatory cytokine expression and reduced insulin-stimulated Akt phosphorylation [124]. In vitro, treatment of A_2A_R-disrupted macrophages with palmitate caused significant increases in pro-inflammatory signaling through JNK p46 and NFκB p65 and pro-inflammatory cytokine expression compared with the treatment of WT macrophages with palmitate [124]. This validates a role for A_2A_R in protecting against macrophage pro-inflammatory activation, thereby adipose tissue dysfunction. In support of this, treatment of obese mice with an A_2A_R agonist caused a significant decrease in adipose tissue macrophage infiltration and improvement in glucose homeostasis [125]. As such, the A_2A_R signaling pathway plays a defensive role in protecting against adipose tissue inflammation.

A_2A_R also plays an important role in alleviating liver inflammation [126]. In A_2A_R-disrupted mice, the expression of pro-inflammatory cytokines in the liver was increased. In contrast, activation of A_2A_R by a selective agonist prevented the accumulation of pro-inflammatory factors and liver damage [126]. Furthermore, the study by Alchera E et al. demonstrated that A_2A_R stimulation inhibited NASH development through inhibiting IL-17-induced JNK-dependent lipotoxicity and Th17 cell expansion [127]. Considering that A_2A_R is expressed in various types of cells, there was a need to address the extent to which the A_2A_R in macrophages plays a protective role in NAFLD. Indeed, this was addressed by Cai et al. In the study by Cai et al., myeloid cell-specific A_2A_R-deficient mice revealed increased severity of HFD-induced hepatic steatosis and inflammation [53]. In macrophage–hepatocyte coculture systems, A_2A_R-deficient macrophages showed increased pro-inflammatory activation and enhanced fat deposition in primary hepatocytes [53]. This validated the importance of A_2A_R-driven macrophage factors in alleviating hepatocyte metabolic and pro-inflammatory responses as key events of NAFLD. It should be noted that A_2A_R-driven macrophage factors also are expected to alleviate dysregulated adipocyte metabolic and pro-inflammatory responses, thereby generating distal effects on the liver to alleviate NAFLD phenotype. This speculation, however, needs to be validated by future investigation. Nonetheless, A_2A_R functions to suppress liver inflammation and lipogenesis, thereby playing a protective role in NASH development.

#### 4.4.5. cGAS-cGAMP-STING-IRF3 Signaling Pathway

Under the stimulation of cytosolic DNA, cyclic GMP-AMP (cGAMP) synthase (cGAS) catalyzes the synthesis of cGAMP from ATP and GTP. As a second messenger, cGAMP binds and activates STING (an important molecule of innate immunity), which in turn activates TANK-binding kinase 1 (TBK1), thereby stimulating interferon regulatory factor 3 (IRF3) to induce the expression of type I IFNs. This pathway participates in inflammation and metabolic responses [128,129]. For example, in HFD-induced obese mice, the expression of cGAS and STING in adipocytes and macrophages were significantly increased. Concurrently, the phosphorylation of TBK1, NFκB p65 and IRF3, as well as the production of TNF-α in adipose tissue were also significantly increased [130]. This suggested that the activation of cGAS-STING signaling pathway in both adipocytes and macrophages contributes to obesity-induced adipose inflammation and metabolic disorder [130]. Disulfide-bond A oxidoreductase-like (DsbA-L) functions to alter intracellular cGAMP, thereby STING activation. This likely accounts for the development of fatty liver [130]. Interestingly, although intracellular (endogenous) cGAMP is involved in pro-inflammatory responses [128,129], exogenous cGAMP exerts cell-type-specific anti-inflammatory effects distinctly from endogenous cGAMP and STING activation. For instance, treatment of an HFD-mouse model with exogenous cGAMP ameliorated diet-induced pro-inflammatory responses and metabolic dysregulation in the adipose tissue [131], which was accompanied by decreased severity of hepatic steatosis and inflammation. Because of this, the cGAS-cGAMP-STING signaling pathway appears to be involved in obesity-associated adipose inflammation, as well as NAFLD phenotype.

Indeed, STING signaling pathway critically regulates NAFLD progression [54,56]. For example, STING expression in liver sections was increased in HFD mouse and NAFLD patients [55,56]. STING was mainly expressed in macrophages, disruption of STING from macrophages was shown to decrease the severity of liver inflammatory responses and fibrosis in NASH mouse models [56]. Moreover, STING activation appears to be sufficient and necessary for inducing liver steatosis and inflammation. As supporting evidence, the study by Yu et al. indicated that treatment of WT mice, but not STING-disrupted mice, with 5,6-dimethylxanthenone-4-acetic acid (DMXAA, a STING agonist) significantly increased hepatic levels of triglycerides and pro-inflammatory cytokines including TNFα and IL-6 [54]. Although the effect of DMXAA treatment is not limited to STING activation, the validation that hepatocyte production of mtDNA in response to excessive fat deposition underlies how Kupffer cells are activated by hepatocyte factor(s) to contribute to liver inflammation provides new insights into the inflammatory mechanisms of NAFLD/NASH [54]. Interestingly, STING in endothelial cells, although not specifically in adipocytes, also was involved in inflammation and metabolic abnormality. Upon treatment with palmitic acid (PA), the mitochondria in human aortic endothelial cells (HAECS) were damaged and mtDNA leakage into the cytosol. The latter activates cGAS, STING and IRF3, which translocated to the nucleus and mediated endothelial inflammation [132]. STING-deficient mice showed reduced endothelial inflammation (in adipose tissue), alleviated IR and glucose intolerance [132], suggesting that it appears to be viable to develop STING inhibitors for NASH management.

As a critical downstream signaling molecule of the cGAMP-STING pathway, IRF3 is also involved in regulating adipose inflammation and maintaining systemic glucose and energy homeostasis [133]. For instance, IRF3 expression was upregulated in adipocytes of obese mice. Upon IRF3 disruption in HFD-mice, inflammation within adipose tissue and the weight of adipose tissue and liver were significantly decreased while glucose uptake in adipose tissue was enhanced. In addition, adipose tissue and liver of IRF3-disrupted mice also revealed modest increases in insulin sensitivity [133]. Hence, it is likely that the IRF3 in adipose tissue generates factors that function to alter liver metabolic dysfunction.

#### 4.4.6. Circadian Regulation

Circadian clocks located in tissues and cells drive daily rhythms and are correlated with many physiological processes including inflammation and metabolism [103]. As key cells of inflammation in obesity, macrophages contain cell-autonomous circadian clocks to regulate macrophage inflammatory responses including rhythms of cytokines secretion [134,135]. For instance, Period1 (Per1) and Period2 (Per2) are vital negative regulators of the circadian clock mechanism and function [136]. In HFD-mice, myeloid cell-specific Per1/2 disruption exacerbated adipose tissue inflammation and dysfunction, impaired adipocyte functions and increased IR and glucose intolerance [103]. In addition, Per1/2-disrupted macrophages showed a much higher pro-inflammatory activation, which was indicated by increased JNK1 and NFκB p65 phosphorylation and IL-1β and TNF-α expression under the stimulation of LPS [103]. Because of this, dysregulation of the macrophage circadian clock, e.g., Per1/2 disruption, is a critical factor through which over-nutrition induces macrophage pro-inflammatory activation, thereby leading to adipose tissue inflammation, hyperglycemia and IR [103]. As such, circadian regulation is a protective factor for adipose metabolic dysfunction.

BMAL1, known as Arntl, is also an important regulator of the circadian clock mechanism. It has been shown that adipocyte-specific Arntl-disruption induced adipocyte hypertrophy and dramatically increased body weight and plasma concentrations of saturated fatty acids [137]. BMAL1/CLOCK heterodimeric proteins are recognized as critical components of cellular circadian rhythm generation [138,139], which appear to be involved in liver disorders under obesity. For example, homozygous CLOCK mutant-obese mice exhibited hyperlipidemia, hepatic steatosis and hyperglycemia and insufficient compensatory insulin production (a hallmark of type 2 diabetes mellitus) [140,141]. Similarly, upon BMAL1 disruption in mice, the plasma adiponectin and leptin concentrations and gluconeogenesis in the liver were significantly increased relative to the control [142]. Although there was no literature concerning the effect of adipocyte-specific disruption of circadian clock genes on the liver, disruption of BMAL1 and CLOCK in mice increased hepatic steatosis and gluconeogenesis. This suggests that BMAL1 and CLOCK appear to play a protective role in regulating liver glucose and lipid metabolism.

### 4.5. Dysregulation of Adipocyte-Macrophage Crosstalk in Obesity-Related WAT Inflammation

As two key cell types of WAT, the interactions of adipocytes and macrophages have been implicated to critically regulate adipose tissue inflammation and metabolic function. In fact, recent advances have demonstrated the roles of factors from the two type cells in regulating the inflammatory and metabolic responses of each other. On the one side, dysfunctional adipocytes produce factors to act on macrophages in adipose tissue and promote the pro-inflammatory responses of macrophages. For instance, the miR-34a expression in adipose tissue is significantly increased during the progression of obesity. When miR-34a was ablated in adipose tissue, obesity-induced metabolic dysfunction was blunted in both the adipose tissue and the liver. As a mediator of adipose inflammation, adipocyte-derived miR-34a was transported into the adjacent macrophages, where miR-34a drives the polarization of macrophages toward pro-inflammatory M1 cells from anti-inflammatory M2 cells [86]. On the other side, macrophages in adipose tissue also produce exosomes, which in turn are involved in the regulation of IR of adipocytes. For example, adipose tissue macrophages (ATMs) from obese mice expressed significantly higher intracellular levels of miR-155. ATM-derived miRNAs (especially miRNA 155) are transferred into adipocytes and hepatocytes (two types of insulin target cells) and impair the insulin sensitivity of these two cells [143]. Therefore, ATM-derived exosomal miRNA critically regulates liver insulin sensitivity, which is also expected to generate profound consequences on liver DNL, thereby hepatic steatosis.

**Figure 2:**
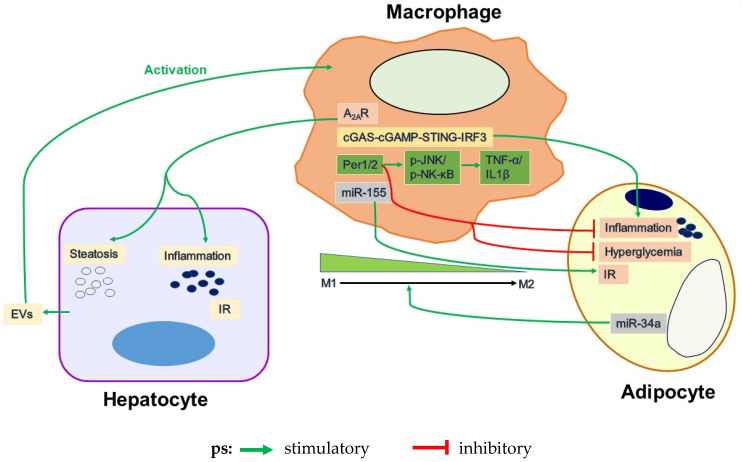
Dysfunctional intercellular signaling within macrophages, hepatocytes and adipocytes in the pathogenesis of obesity-related NAFLD. A_2A_R, STING and Per1/2 are predominantly expressed in macrophages. Increased A_2A_R and/or Per1/2 in macrophages functions to protect whereas up-activated cGAS-STING signaling pathway in macrophages functions to aggravate fat deposition and inflammatory responses in hepatocytes. Furthermore, overexpression of Per1/2 in macrophages inhibits inflammatory responses in adipocytes. MiR-155 from macrophages aggravates insulin resistance. In contrast, miR-34a from the adipocytes promotes the polarization of macrophages toward M2 cells from M1 cells. Moreover, stressed hepatocytes release increased numbers of EVs and promote macrophage activation and accumulation. ps: EVs: extracellular vesicles.

As described above, the activation status of macrophages in white adipose tissue is tightly associated with not only adipose tissue function, but also hepatic steatosis and inflammation. This role played by macrophages is summarized in Figure 2.

## 5. Perspective and Future Directions

The prevalence of obesity remains high not only in general populations, but also in children. Because of this, the incidences of obesity-associated NAFLD and NASH are expected to increase continuously. While there still lacks an effective treatment for NASH, preventative and/or therapeutic approaches for managing obesity are of particular importance in reducing or alleviating the development and progression of NASH, thereby NASH-related terminal liver diseases. In terms of pharmacological approaches, there is a critical need to develop new agents to improve adipose tissue dysfunction in obese subjects, including agents that primarily target adipocytes to decrease stress-induced adipocyte necrosis or neutralize pro-inflammatory and pro-NAFLD/NASH factors released by apoptotic adipocytes. While various signaling pathways involved in adipose tissue inflammation also are involved in liver inflammation, it is necessary to target key signaling molecules of the pathways to additively or synergistically reduce adipose tissue and liver inflammation with the hope to effectively manage or correct dysfunctional crosstalk between adipose tissue and the liver, thereby providing improved efficacy at treating obesity-associated NAFLD. Based on recent advances, approaches that prevent or block adipocyte death-initiated macrophage activation may offer a new strategy for managing NAFLD while the approach appears to be promising in managing alcohol-induced fatty liver disease [59]. Dietary approaches that alter circulating levels of branched chain amino acids appear to also modify viscera fat lipolysis, thereby liver steatosis [144]. As discussed before, BAT, unlikely WAT, is shown to have a reverse correlation with NAFLD [28]. This implies that approaches targeting BAT to increase thermogenesis and improve BAT functions are also likely to serve the effective management for NAFLD. During obesity, adipose tissue autophagy is increased, and autophagy inhibition ameliorates NAFLD phenotype [145]. This suggests that therapeutic agents that inhibit WAT autophagy is viable for managing NAFLD. Excitingly, adipose tissue-derived stem cells are shown to reverse NAFLD [146]. This likely also offers a non-classic adipose tissue-based approach for managing NAFLD.
